# Innovative Non-Invasive and Non-Intrusive Precision Thermometry in Stainless-Steel Tanks Using Ultrasound Transducers

**DOI:** 10.3390/s24113404

**Published:** 2024-05-25

**Authors:** Ahmed Bouzid, Saad Chidami, Tristan Quentin Lailler, Adrián Carrillo García, Tarek Ould-Bachir, Jamal Chaouki

**Affiliations:** 1 MOTCE Laboratory, Department of Computer Engineering, Polytechnique Montréal, Montreal, QC H3T 1J4, Canada; tristan-quentin.lailler@polymtl.ca; 2 PEARL Laboratory, Department of Chemical Engineering, Polytechnique Montréal, Montreal, QC H3T 1J4, Canada; saad.chidami@polymtl.ca (S.C.); adrian.carrillo-garcia@polymtl.ca (A.C.G.); jamal.chaouki@polymtl.ca (J.C.)

**Keywords:** thermometry, water monitoring, ultrasound instrumentation, time-of-flight measurement, non-destructive testing, non-invasive testing

## Abstract

Measuring temperature inside chemical reactors is crucial to ensuring process control and safety. However, conventional methods face a number of limitations, such as the invasiveness and the restricted dynamic range. This paper presents a novel approach using ultrasound transducers to enable accurate temperature measurements. Our experiments, conducted within a temperature range of 28.8 to 83.8 °C, reveal a minimal temperature accuracy of 98.6% within the critical zone spanning between 70.5 and 75 °C, and an accuracy of over 99% outside this critical zone. The experiments focused on a homogeneous environment of distilled water within a stainless-steel tank. This approach will be extended in a future research in order to diversify the experimental media and non-uniform environments, while promising broader applications in chemical process monitoring and control.

## 1. Introduction

The monitoring temperature in chemical reactors is of extreme importance since it plays a crucial role in the determination of reaction rates, selectivity, and product quality. By closely monitoring the temperature, optimal operating conditions could be ensured while preventing unwanted side reactions and product degradation. It also allows for maintaining the overall efficiency of the reactor [1,2]. However, performing temperature measurements in chemical reactors could be a challenging task due to the harsh and complex nature of the reactor environment. Traditional measurement techniques are often invasive and involve the physical insertion of probes or sensors inside the reactor, which can lead to the disruption of the flow patterns, alter the properties of the system, and introduce a potential contamination or create safety risks [3,4].

Non-invasive measurement techniques address these challenges by being performed without a direct contact with the reactor contents, thus eliminating the need for intrusive probes. This helps in preserving the integrity of the system, minimizing the risk of contamination, and ensuring the safety of the operators. Furthermore, non-invasive techniques enable real-time monitoring without disturbing the ongoing reactions or flow dynamics. This provides a more accurate representation of the actual conditions within the reactor and allows for immediate adjustments or interventions when necessary.

Among the variety of existing non-invasive techniques, acoustic resonance spectroscopy involves analyzing the resonant frequencies of a sample to determine properties such as its density and viscosity. The measurement is realized by the excitation of the sample with acoustic waves and analyzing the response, obtaining non-invasive measurements [5,6,7]. A second method, infrared thermometry, uses the principles of infrared radiation to measure the temperature. Infrared cameras or pyrometers are used to capture the thermal radiation emitted by an object, providing non-contact temperature measurements [8]. A third method is the use of optical fiber sensors for temperature and strain measurements; they operate based on the changes in light transmission through an optical fiber due to temperature variations or mechanical deformations [9]. Magnetic resonance imaging (MRI) is another powerful imaging technique that can provide temperature and flow information within a sample. By utilizing the phenomenon of nuclear magnetic resonance, MRI can generate detailed spatial maps of temperature and fluid flow patterns [10]. Lastly, optical density measurement methods such as absorption spectroscopy and light scattering techniques can be used to measure the density of fluids or suspensions; these techniques rely on the interaction of light with the sample to extract density-related information [11].

In the case of the assessment of water temperature within a stainless-steel container, the method employed relies on acoustic PFT (Peak Flight Time) measurements. This method, as opposed to the previously mentioned techniques, is based on the distinctive attributes of ultrasound, which is pertinent to our specific application. Ultrasound offers several notable advantages, including its non-destructiveness, non-intrusiveness, waste-minimization, non-hazardous nature, being pluggable, real-time property, and adaptability to challenging industrial environments [12]. In contrast to X-ray and microwave methodologies, ultrasound proves to be a secure, cost-effective alternative, and it is particularly safe with regard to the risks posed to the health of operators. Diverging from acoustic spectroscopy, the utilization of PFT-based acoustic measurements provides a real-time and an economically viable solution.

Ultrasound has demonstrated its effectiveness in the scientific literature and industrial applications for measuring distance [13], flow velocity in pipes [14], and fluid levels in containers [15]. Ultrasound is commonly employed in medical applications, particularly for temperature imaging to diagnose carotid artery disease [16] and monitoring temperature during medical treatments like high-intensity focused ultrasound [17,18]. In certain industrial applications, furnaces are frequently utilized, and it is possible to visualize temperature distributions using acoustic tomography to ensure combustion stability [19,20,21]. Measuring temperature through ultrasound becomes challenging when the acoustic wave passes through media with significantly different acoustic impedances, such as metal–air coupling. In such cases, it is preferable to integrate ultrasound sensors into tanks or pipes [22]. However, this alternative comes with several drawbacks, including contamination risks, maintenance and transducer replacement difficulties, and the necessity to adapt the tanks or pipes to accommodate the transducers.

M. Schwarz and B. G. Zagar in [23] proposed a method for the non-intrusive 2D reconstruction of water temperature using an array of ultrasound transducers. The method, although invasive, was tested in an acrylic glass reactor. Through their experiments conducted at temperatures ranging from 18 to 30 °C, they demonstrated an achievable uncertainty of 0.5 °C. In [24], Miklós Lenner et al. demonstrated a non-invasive temperature measurement of water using ultrasound within a limited temperature range (23 °C to 45 °C).

The majority of the methods mentioned in the scientific literature are often invasive and/or intrusive, and they typically handle a relatively narrow temperature range tested in plastic reservoirs, thus limiting their applicability [23,24,25,26]. Building upon these research efforts, this paper introduces an innovative method that aims to extend the utility of non-invasive and non-intrusive ultrasound-based temperature measurements to a much wider range, covering temperatures from 28.8 to 83.8 °C utilizing a stainless-steel reactor. The expanded temperature scope enhances the versatility and practicality of the method, making it applicable to a wider range of real-world scenarios and industrial processes.

In this paper, we introduce a cost-effective, non-intrusive, and non-invasive method for measuring water temperature along the path of an acoustic wave within a stainless-steel container using just one pair of transducers. The proposed approach covers a wide temperature range and relies on precise acoustic peak flight time (PTF) measurements in water, resulting in a more accurate temperature determination. To validate this method, a reference temperature measurement system was designed and used. This initial setup was then expanded to eight pairs of transducers to create an array, and by employing image reconstruction techniques, temperature tomography was performed, providing estimates of the temperature distribution.

The key contributions of this paper can be summarized as follows:

1. The paper proposes an innovative technique based on ultrasonic measurements for non-invasive and non-intrusive thermometry. This method offers an alternative to traditional invasive approaches and provides more accurate and instantaneous measurements.
2. It presents a methodology for obtaining a temperature measurement polynomial adapted to industrial setups via a detailed calibration procedure.
3. The experimental results presented in the paper demonstrate the accuracy of the developed ultrasonic-based technique. This highlights its potential in improving measurement precision in complex reactor environments.
4. By integrating ultrasonic sensors within the reactor setup, the proposed method allows for the continuous and non-invasive/non-intrusive monitoring of temperature across a wide temperature range in a stainless-steel reactor.
5. The paper shares valuable lessons learned from the experimental setup, including important considerations such as sensor placement, signal processing techniques, and system calibration.

The remainder of this paper is organized as follows. Section 2 provides a comprehensive overview of the materials and methods employed in this study, outlining the experimental setup, instrumentation, and the measurement technique used for ultrasonic thermometry. Section 3 presents the detailed experimental methodology used to validate the solution, and it emphasizes the calibration, the experimental setup, and the experimental protocol. Section 4 presents the experimental results obtained through the application of the proposed method, displaying the accuracy and reliability of the measurements. Finally, Section 5 offers a brief conclusion, summarizing the main contributions of this study and discussing potential future directions for further advancements in this field.

## 2. Materials and Methods

### 2.1. Acoustic Time-of-Flight and Peak Flight Time Measurement Principles

The proposed methodology considers acoustic impedance properties in the propagation path, taking into account the transmission of ultrasonic waves from an ultrasonic transducer through stainless steel, to water, and back through stainless steel to the receiving ultrasonic transducer. This method is influenced by the time-of-flight (ToF) of sound in distilled water, a critical factor in the study of acoustic wave propagation. Historically, researchers have developed polynomial models that link the speed of sound in water to temperature, with prominent models such as Del Grosso, Greenspan, Wilson, and others [27]. These models offer valuable insights into the link between temperature and sound velocity in water, serving as the foundation for our temperature measurement methodology.

The ToF measurement principle relies on the assessment of the time taken for an ultrasonic signal to travel from the transmitter to the receiver. The acoustic impedance of the media through which the signal propagates, particularly the stainless-steel reactor walls and the distilled water within, significantly influences this measurement. Primarily, acoustic wave transmission was noticed when the main medium was air, where the impedance is significantly smaller compared to stainless steel (413 Ry versus 45.7 MRy, respectively). However, in this study, the problem was not significantly evident because water, the primary medium, has an impedance of 1.48 MRy, which is closer to that of stainless steel. Acoustic impedance acts as a crucial intermediary in determining the celerity of sound within the medium, enabling temperature assessment. The empirical polynomial model used in this study provides a mathematical expression that correlates sound speed *c* (in m/s) in water with temperature variations (in °C), which is expressed as described by Del Grosso following [28]:

(1) $$c=\sum_{k=0}^{5}a_{k}T^{k},$$

where the coefficients $a_{0}$ to $a_{5}$ are defined in Table 1:

Equation (1) and its coefficients described in Table 1 concern the relationship between the speed of sound and the temperature of pure water (no salinity) at normal atmospheric pressure. However, the main medium considered in this research was pure water without neglecting the stainless-steel walls and the Ethyl 2-cyanoacrylate glue which significantly affect the polynomial. The speed of sound is also related to pressure and the salinity of water as adopted by UNESCO as the international standard and modified by Wong and Zhu [31]. The pressure was not considered in this study since it had no significant impact on the results. The transducers were placed at 7 cm below the water surface, and at this depth, the pressure increased by 0.68 kPa, which is insignificant compared to the daily atmospheric pressure variation of 3 kPa. Although many solutions for measuring temperature using ultrasound rely on measuring the speed of sound [32], this study’s research design utilized Acoustic Peak Flight Time (PFT) measurement. This method can be understood as the time taken for the peak of the acoustic signal to travel.

It is worth noting that temperature measurements via sound velocity measurements using time of flight is not feasible in the present case since the beginning of the A-Scan often has a low SNR where the signal is lost in the noise from waves transmitted by metal and other sources, making time of flight detection challenging. Furthermore, the use of the polynomials presented in the literature is not applicable due to the significant modification of this polynomial by our experimental setup. Standard polynomials are rendered inapplicable due to the fact that transducers are not fully immersed in water; instead, a glue–metal interface exists between the water and each of the transducers, resulting in substantial alterations to the coefficients. The aim of this research was also to propose a method for extracting a polynomial specific to the industry-used setup through a detailed calibration process as outlined in this article.

### 2.2. Signal Generation and Processing

#### 2.2.1. Ultrasonic Transducers and Working Frequency

**Figure 1:**
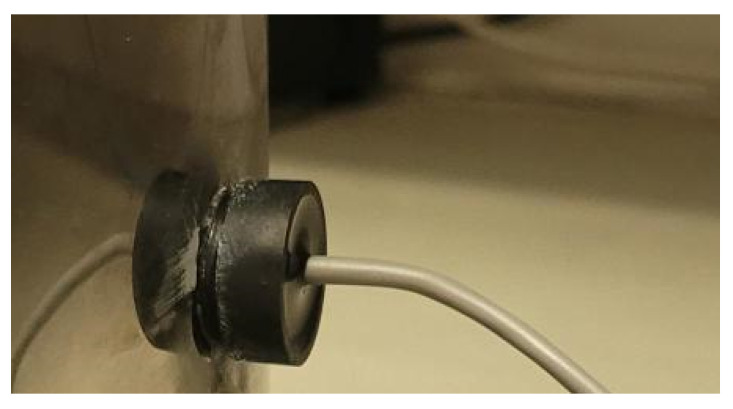
Photograph of H2KMPYA1000600 ultrasonic transducer attached to the stainless-steel reactor.

The selection of appropriate ultrasonic transducers was a critical aspect of this research. Several key parameters, including frequency, diameter, thickness, material composition, impedance, and electrical model, were meticulously considered in the decision-making process. After a comprehensive evaluation, the ultrasonic transducer H2KMPYA1000600 model (see Figure 1) was identified as the most suitable for this study. Table 2 outlines the key parameters of the ultrasonic transducer used. This choice aligns with the requirements of the research objectives and ensures optimal signal transmission and reception characteristics.

To ensure the optimal working frequency for the temperature measurement within the stainless-steel chemical reactor, a series of empirical tests were conducted. Following the installation of the selected transducers in the reactor walls, a frequency sweep was performed. The tests revealed that the most effective frequency, when the reactor is filled with water, was 1007 kHz. This frequency was selected as it exhibited optimal signal propagation characteristics and minimal interference.

#### 2.2.2. Ultrasound Driver and Receiver

The ultrasound driver, a key component of the experimental setup, requires the consideration of various parameters including voltage, current, waveform, number of cycles, and frequency. Voltage and current values are adjusted to achieve a decent signal strength and proper wave propagation. The waveform design and number of cycles play an essential role in signal integrity. Frequency, a critical parameter, is chosen to maximize the signal-to-noise ratio and minimize interference.

**Figure 2:**
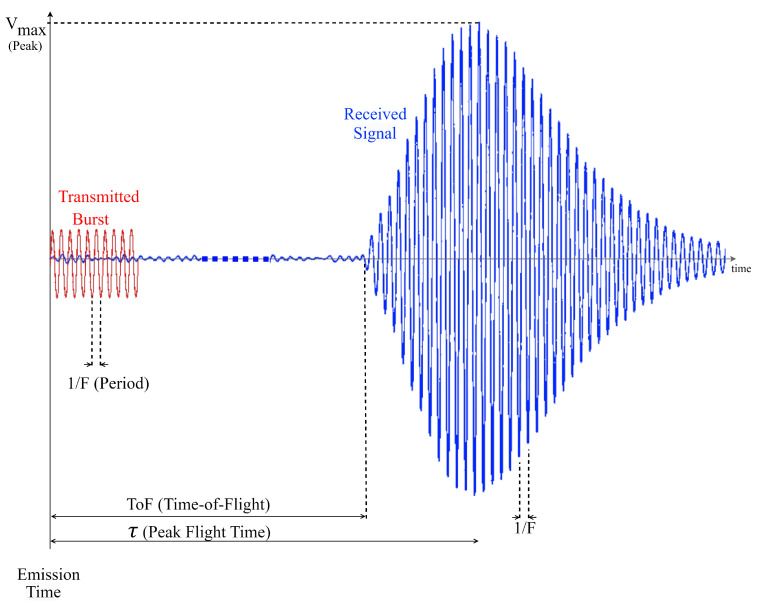
Characteristics of received Ultrasonic A-Scan signal (in blue) when emitting 10 sine waves (in red).

The ultrasound receiver configuration includes essential elements including preamplification and a low-pass filter for anti-aliasing and eliminating undesired high-frequency components. These components enable the precise measurement of the time taken for the ultrasonic wave to cross the reactor medium and receive a coherent ultrasonic A-Scan signal as detailed in Figure 2.

Data collection required the integration of the Analog Discovery 2 (AD2) within the experimental setup, with a parameter configuration controlling signal generation and reception for reliable data acquisition. Key parameters included the utilization of a 10Vpp (Peak-to-Peak Voltage) signal burst, the usage of a sine wave waveform for signal fidelity, the inclusion of 10 cycles per burst, operation at a frequency of 1007 kHz to optimize SNR while addressing potential interference, the adoption of an Analog-to-Digital Converter (ADC) sampling time of 33.33 MSPS (Mega Samples Per Second) for accurate analog-to-digital signal conversion, and the transmission and reception of a total of 10 signal bursts per temperature setpoint for further statistical analyses. The resolution of the ADC was 14-bit, and the voltage resolution was set to 0.32 mV, the smallest achievable with the Analog Discovery 2.

The automation of the signal generation and data collection process was achieved through a custom script developed in WaveForms software version 3.19.5.

#### 2.2.3. Field Programmable Analog Array (FPAA)

**Figure 3:**
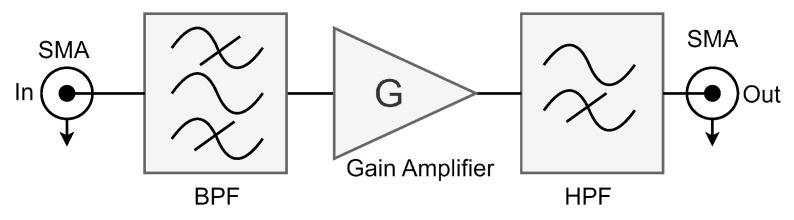
Rx Analog Front-End design and implementation on FPAA. Analog preprocessing step for anti-aliasing and amplification.

To create an interface between the receiving transducer and Analog Discovery 2, an FPAA (AN221E04 from Anadigm) was incorporated into the experimental setup as an Analog Front-End (AFE). The FPAA served multiple crucial functions, including signal amplification with a gain of 26.8 and band-pass filtering centered at 1007 kHz. Extensive testing revealed the necessity of implementing a high-pass filter immediately before the FPAA output to effectively eliminate the 60 Hz power line noise (as shown in Figure 3). The hardware resources required for the FPAA design were 15 capacitors and 4 operational amplifiers.

Careful instrumentation setup guarantees precise and reliable data generation and acquisition in stainless steel chemical reactors.

### 2.3. Reference Temperature Measurement System

**Figure 4:**
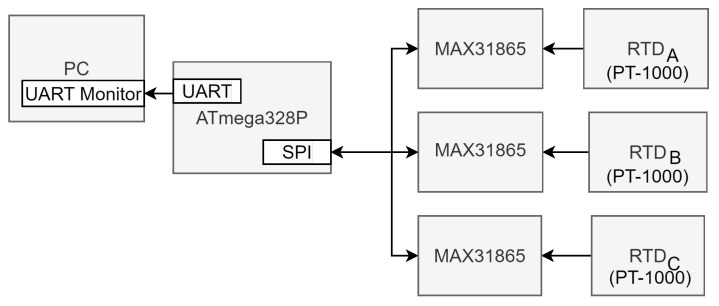
Block diagram illustrating the design of the reference temperature measurement system utilizing three RTDs for precise temperature monitoring.

To establish a reliable reference temperature measurement system for comparative purposes, three Resistance Temperature Detectors (RTDs) of type PT-1000 were employed. These RTDs were interfaced with three MAX31865 conditioners/preprocessors via the Serial Peripheral Interface (SPI) communication protocol (see Figure 4). The data processing and temperature computation were carried out by an ATmega328P microcontroller that transmits the temperature values serially to a PC for data logging and analysis.

The foundation of this temperature measurement system lies in the application of the Callendar–Van Dusen equation as defined in the IEC751 standard. This equation establishes a relationship between temperature (*T*) and the resistance (*R*) of the RTD as follows:

(2) $$R=R_{0}\left(1+3.9083\;10^{-3}T-5.775\;10^{-7}T^{2}\right)$$

where $R_{0}$ represents the resistance of the RTD at 0 °C.

### 2.4. The Proposed Temperature Measurement Method

**Figure 5:**
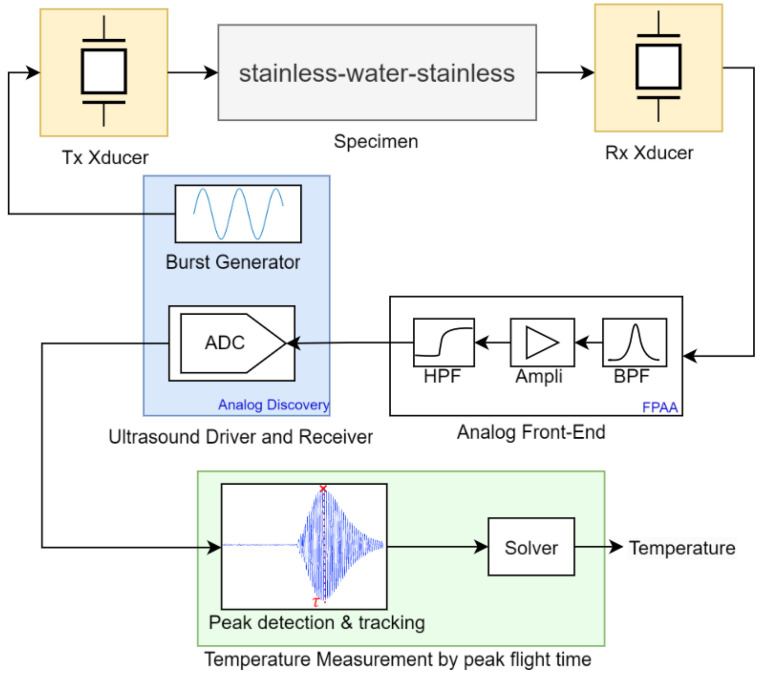
Block diagram representing the process for estimating water temperature non-invasively and non-intrusively in a stainless-steel reactor using ultrasound transducers and relying on the PFT measurement method.

This section explains the methodology used for temperature assessment within a chemical reactor containing water, utilizing ultrasonic transducers positioned outside the reactor. The method covers two principal phases: the initial calibration, performed during system installation, and the subsequent temperature measurement. Preliminary experiments confirmed that wave velocity is considerably affected by the steel and the glue with respect to the temperature. The polynomials presented in the literature concern the relationship between the speed of sound and temperature when transducers are submerged in pure water. In this study, glue–steel–water–steel–glue coupling was considered; hence, a meticulous calibration phase was carefully considered prior to all experiments. The following section explains the detailed algorithm of the proposed method:

- Define the Constants: The temperature measurement methodology requires the definition of key constants. These constants include the buffer size of the ADC of Analog Discovery 2, the frequency of the transmitted signal, the sampling frequency, and the number of bursts transmitted per temperature setpoint.
- Calibration: The calibration process of the method mainly consists of determining the polynomial constants that establish the relationship between the PFT (τ) and temperature (*T*), represented as τ=f(T). Within the calibration phase, the methodology capitalizes on the assumption that the rising time of an A-Scan remains invariant with respect to temperature variations (which was experimentally verified) but specific to the setup. The calibration process has multiple steps, starting with the measurement of a reference temperature ($T_{ref}$) for the water performed by a reference measurement system. The calibration procedure is followed by the transmission and reception of ten bursts of ten sinusoids each, all characterized by predefined parameters. The approach to extract τ (the time at which the signal reaches its maximum) is restricted to the detection of the same peak as the previous measurement. Experiments have shown the potential for misalignment in peak detection, resulting in temperature bias, and this approach serves to minimize divergence. Note that this technique operates under the condition that the temperature of the process does not increase by more than 3 °C per sample period, which demonstrates the rapidity of the method. On the contrary, exceeding this condition is hardly conceivable in real-life scenarios. The final step of the calibration process involves determining the coefficients of the polynomial that best fit the measured PFTs (τ) with the reference temperatures. Note that this calibration procedure is only performed when there is a change in the setup: such as changing reactors, transducers, or attachment materials.
- Temperature Measurement Procedure: The temperature measurement involves transmitting and receiving bursts, measuring τ, and then calculating the temperature by solving the polynomial equation defined using the coefficients obtained during the calibration process. Figure 5 summarizes the temperature measurement method using the PFT technique.

## 3. Experimental Methodology

### 3.1. Calibration of RTD Sensors

The calibration process of the temperature sensors ensures the accuracy of the reference temperature measurement system. As per the IEC751 standard, the procedure aims to determine the resistances at 0 °C ($R_{0}$) for each of the three PT-1000 RTD sensors (RTDA, RTDB, and RTDC), which consists of the following steps:

- Filling a container with crushed ice.
- Adding water to the container until it reaches approximately 1 centimeter below the top of the ice.
- Immersing the RTD sensors in the middle of the container.
- Stirring the ice bath for one minute to ensure temperature uniformity.
- Measuring the resistance of the RTDs.

The calibration process gives the following resistance values: 999.56 Ω for RTD_A_, 999.95 Ω for RTD_B_, and 999.81 Ω for RTD_C_.

### 3.2. Experimental Setup

**Figure 6:**
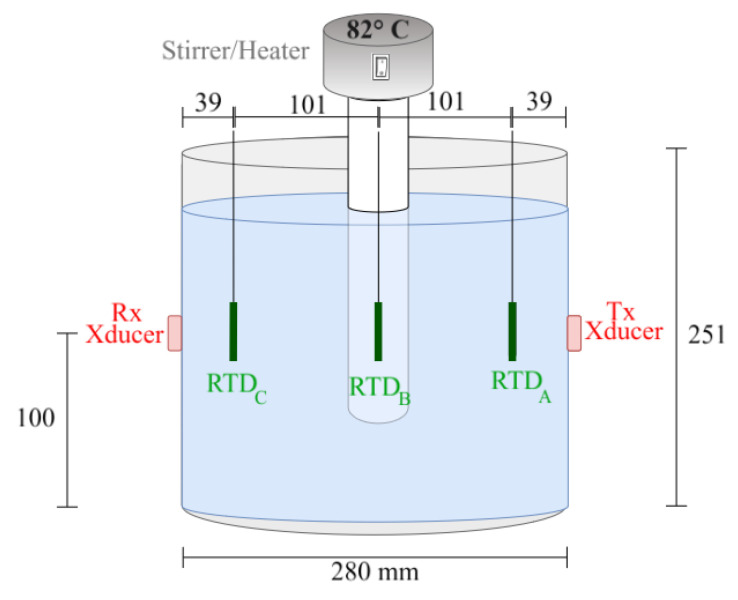
Schematic diagram illustrating the reactor’s dimensions and the positioning of the ultrasonic transducers and the RTD sensors.

The reactor, in the present case a stainless-steel pot (see Figure 6), has a resonance frequency of approximately 58 kHz, which is far from the operational frequency of 1007 kHz. To optimize wave transmission through the stainless steel, Ethyl 2-cyanoacrylate glue was used as an adhesive and acoustic couplant, which was chosen for its crystalline and incompressible properties.

**Figure 7:**
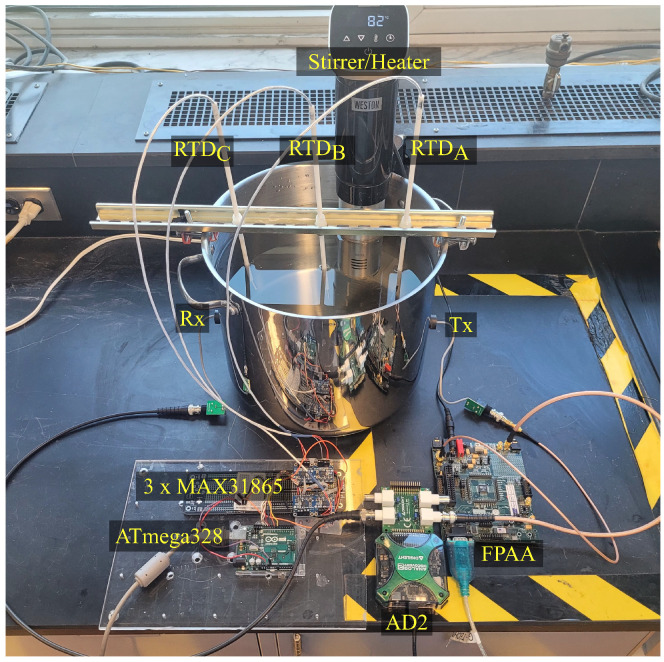
Global view of the experimental setup showing the integrated components for temperature measurement and validation.

To ensure signal fidelity and facilitate connectivity, coaxial cabling integrated with BNC and SMA ports was used. Printed Circuit Boards (PCBs) including BNC ports were designed to connect the transducers with the Analog Discovery BNC adapter board though the coaxial cables. A stirrer equipped with a temperature sensor and heating element was submerged in the water to homogenize temperature distribution (see Figure 7).

### 3.3. Experimental Protocol

The experimental protocol was defined to ensure data acquisition and maintain the integrity of the measurements within the temperature range of 28.8 to 83.8 °C. After filling the water tank with 12.3 L (which, by the end of the experiment, decreased to 11 L due to evaporation), the steps of the experimental protocol included the following:

(i) Setting the temperature setpoint to initiate the experiment within the predefined temperature range of 28.8 to 83.8 °C.
(ii) Activating the stirrer/heater to initiate the homogenization of the water temperature and the controlled heating process.
(iii) Removing air bubbles accumulating on the walls of the reactor and the RTD sensors to eliminate potential acoustic reflections, attenuation, and scattering.
(iv) Temperature stabilization to wait until reaching the setpoint temperature.
(v) Transmitting and receiving ultrasound signals: Ten bursts of ultrasound signals were generated by the AD2 and transmitted by the Tx Xducer (transducer) and then received by an Rx transducer. The choice of using 10 bursts in series is based on the fact that at around 73.2 °C (the point where the wave velocity is maximum according to our setup), there is a high probability of temperature divergence in the calculated values. Therefore, increasing the number of bursts helps to better identify the temperature that converges most accurately.
(vi) Data acquisition: signals are then conditioned by the AFE and acquired by the AD2.
(vii) Cycle continuation: returning to Step (i) to initiate a new cycle and ensure a continuous data acquisition process.

## 4. Results and Discussion

### 4.1. Ultrasound PFT Measurement

**Figure 8:**
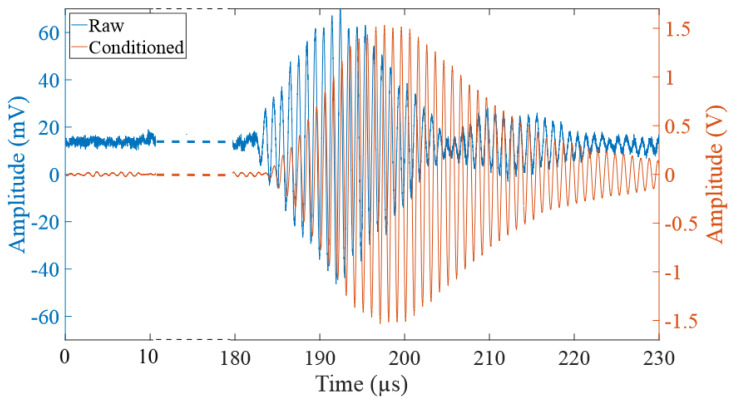
Received raw (blue) and received conditioned (orange) signals at 76.5 °C.

Figure 8 illustrates a portion of the received signal resulting from the transmission of a single burst consisting of 10 sinusoids with a frequency of 1007 kHz and an amplitude of 10Vp-p when the water was heated to 76.5 °C. Consequently, the PFT algorithm detects a peak at 197.62 μs.

The orange signal (from the output of the AFE conditioner) shows an A-Scan that is much stronger than the preceding noise, which indicates a good Signal-to-Noise Ratio (SNR). In contrast, a low SNR would lead to a divergence in the measured temperatures. The signal conditioned by AFE displays a delay of about 1 µs due to the filters; however, the SNR is 35.2 dB, compared to 21.5 dB before conditioning. The raw signal shows a bias due to the 60 Hz power line noise but is canceled by the AFE.

The derived equation *f* that represents the relationship between the peak flight time τ and the reference temperatures *T* measured by the RTDs is a fourth-degree polynomial expression and presented as follows:

(3) $$\begin{array}{c} \tau =f(T)=\;2.14361\times 10^{-4}-6.45467\times 10^{-7}T+7.96744\times 10^{-9}T^{2}\\ \;\;\;\;\;-4.61802\times 10^{-11}T^{3}+1.41077\times 10^{-13}T^{4} \end{array}$$

**Figure 9:**
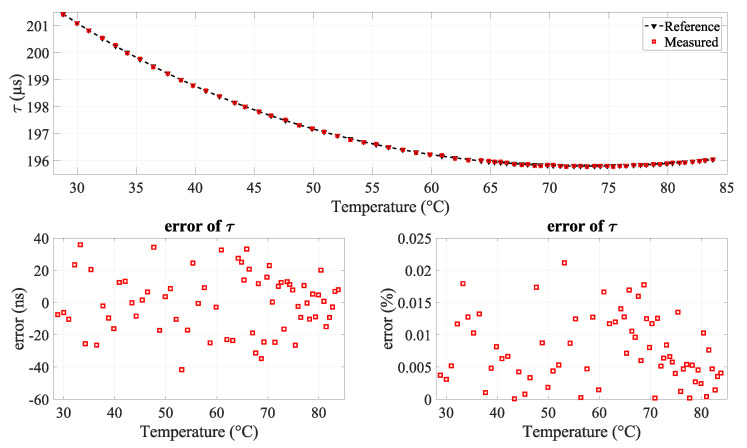
(**Top**) Correlation between measured acoustic rise time, reference acoustic rise time, and true temperature; (**bottom**) error in acoustic rise time (in ns and percentage).

The observed trendline presented in Figure 9 shows a robust correlation between the measured acoustic rise time and the actual water temperature, derived from the reference temperature measurement system spanning a temperature range from 28.8 to 83.8 °C.

The residual errors in nanoseconds (ns) and in percentage (%) associated with the polynomial fitting are presented in the bottom part of Figure 9. The results show a considerable level of accuracy, characterized by an uncertainty of ±42 ns corresponding to 0.021%, while the standard deviation is 18.29 ns. It is also observed that the absolute area of the AScan decrease with increasing temperature.

### 4.2. Temperature Measurement from PFT Method

**Figure 10:**
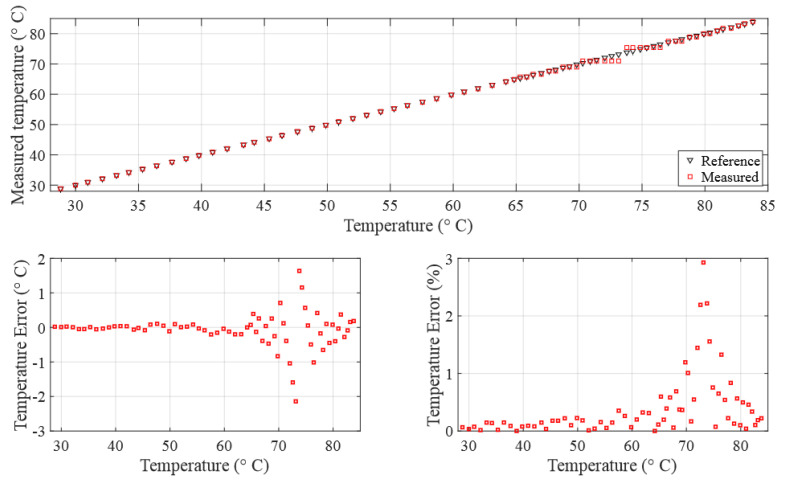
(**Top**) Measured temperature with respect to the true temperature; (**bottom**) error in temperature measurement (in °C and percentage).

Regardless of the promising nature of the findings, it is worth showing the impact of the uncertainties of the rise time measurements on the accuracy of the measured temperature, especially in the region of the turning minimum point at about 73.2 °C. Figure 10 illustrates the temperature measurement derived from acoustic rise time measurements and the solution of Equation (3), which characterizes the relationship between the rise time and temperature.

The plot reveals three regions: the first and third regions display temperature errors below 1%. In contrast, the second region, which we will call the critical zone, spans from 69 °C to 77.5 °C, where temperature errors exceed 1%. The critical zone appears due to the behavior of the fourth-degree polynomial which defines a local minimum near 73.2 °C. Within this critical zone, even minor deviations in rise time measurement result in significant temperature measurement errors and potential divergence. To mitigate this critical zone and enhance measurement precision, one effective approach is to reduce the ADC sampling time.

### 4.3. Refined-Technique Findings

**Figure 11:**
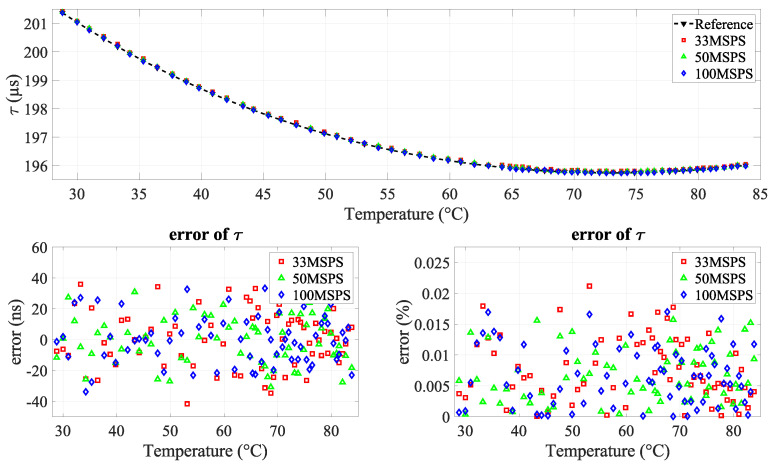
(**Top**) comparison of measured acoustic rise time at different ADC sampling frequencies (33.33, 50, and 100 MSPS) relative to true temperature; (**bottom**) error in acoustic rise time (in ns and percentage).

For the same experimental setup and liquid, experiments were repeated using three different ADC sampling frequencies (33.33, 50, and 100 MSPS). The solution was tested using the previously defined measurement method. As shown in Figure 11, the results support the hypothesis demonstrating an improvement in the precision of rise time measurements, where the standard deviation decreases from 18.29 ns to 16.06 ns.

**Figure 12:**
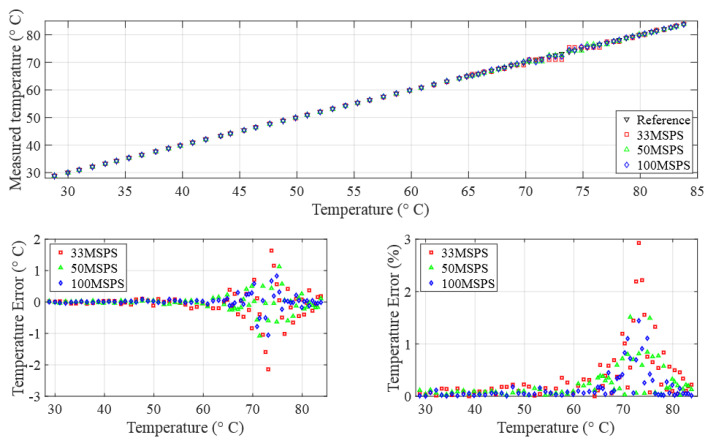
(**Top**) comparison of measured temperatures at different ADC sampling frequencies (33.33, 50, and 100 MSPS) relative to the true temperature; (**bottom**) error in temperature measurement (in °C and percentage).

This improved precision in rise time measurements results in an enhanced accuracy in the measured temperatures, as illustrated in Figure 12. The maximum error, observed within the critical zone, reduces from 2.14 °C (2.92%) to 1.05 °C (1.4%). Furthermore, it is noticeable that the critical zone is narrowed from [69 °C, 77.5 °C] for 33.33 MSPS to [70.6 °C, 75.1 °C] for 100 MSPS. Additionally, the standard deviation of the temperature decreases from 0.42 °C to 0.21 °C.

**Figure 13:**
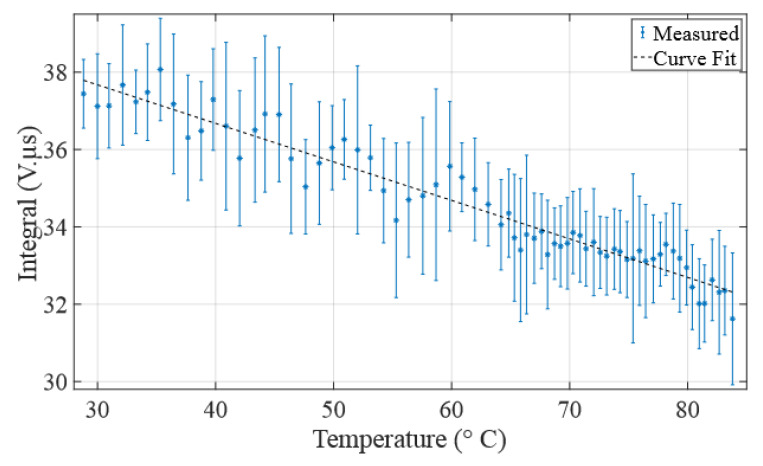
Temperature-dependent variations in A-Scan absolute area: observing how temperature influences the absolute area of the received pulses.

Though experiments, it is observed that the absolute area of the A-Scan is inversely proportional to the water temperature, ranging from 37.7 V·μs to 32.5 V·μs for the temperature range under investigation in this research (see Figure 13). The measurement of this area at temperature *T* is approximated by the trapezoidal integration method of the absolute value of the received signal *f* such that

(4) $$absolute\;area{|}_{T}=\int_{t_{a}}^{t_{b}}\left|f(t)\right|dt{|}_{T}\approx \frac{\delta t}{2}\sum_{k=1}^{N}\left|f(t_{k-1})\right|+\left|f(t_{k})\right|{|}_{T}$$

where $t_{a}$ and $t_{b}$ are the start and end times of the received signal, in this case, the beginning and end of the pulse, respectively. δt is the sampling period, which is constant in this case (10 ns). *N* is the number of signal samples. $f(t_{k})$ represents the voltage of the received signal at the k-th sample. Analog Discovery 2 was set to receive and store data on a buffer of 16,384 samples, which correspond to an observation window of $t_{b}-t_{a}$= 163.84 μs. The starting time of the observation window ($t_{a}$) is fixed to 118.08 μs for all the experiments, and $t_{b}$ is then equal to 281.92 μs.

As shown in Figure 13, this method of temperature measurement based on the absolute area of the signal presents uncertainties. However, if improved, it could to be exploited to automate temperature measurements by combining it with the proposed method since the selection of the right solution from the solver of Equation (3) is not treated in this paper. When the solver proposes two solutions, measuring the absolute area could determine the appropriate side of the polynomial curve.

### 4.4. Validation Experiment

**Figure 14:**
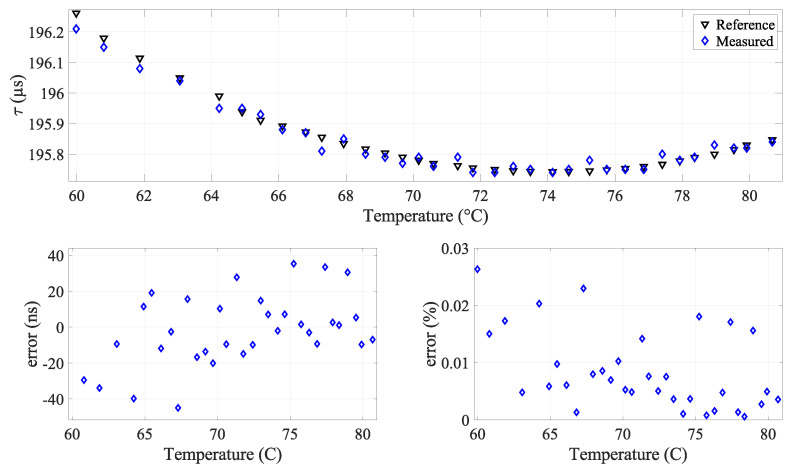
Validation experiment results: (**top**) comparison of measured and reference rise time; (**bottom**) error analysis at 100 MSPS sampling frequency.

**Figure 15:**
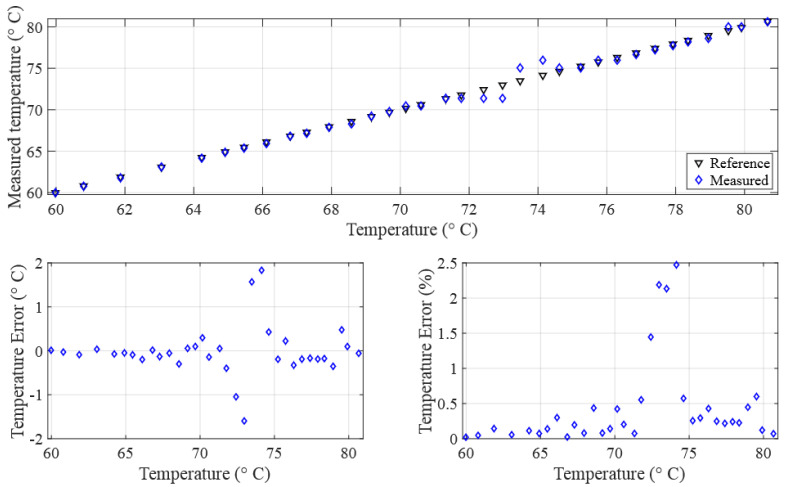
Validation experiment results: (**Top**) comparison of measured and reference temperatures; (**bottom**) error analysis at 100 MSPS sampling frequency.

An experiment was conducted to validate the method by measuring the temperature of pure water. The ADC sampling time was set to 10 ns to maximize the precision. The chosen temperature range spans 60 °C to 83.9 °C. This range was chosen because outside, precision is always guaranteed. The experimental results of acoustic rise time and temperature measurements are presented in Figure 14 and Figure 15, respectively.

The results show a decrease in the level of accuracy in the temperature measurements, with errors remaining below 0.5%. The critical zone is defined within the interval [72.1 °C, 74.4 °C], showing a peak error of 1.8 °C (2.4%).

**Figure 16:**
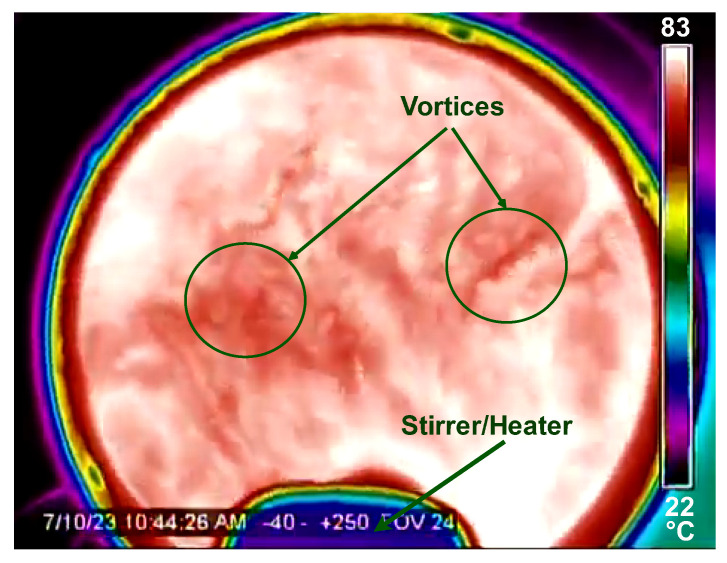
Thermal image captured during water stirring and heating in the reactor at 83 °C setpoint.

It is worth noting that tests in stagnant (inert) water provide significantly more precise results. The experiments were conducted in turbulent water caused by the agitation of the mixer/heater as illustrated in Figure 16.

## 5. Conclusions

This study presents a novel approach to the ultrasound-based temperature measurement of a large temperature range within a stainless-steel tank. By means of the PFT (Peak Flight Time) measurement technique and a rigorous calibration process, we achieved precise temperature estimations in stagnant and turbulent water conditions. Our work validates the efficacy of establishing a polynomial model for a specific experimental setup by means of meticulous calibration procedures. This research significantly contributes to advancements in non-invasive and non-intrusive temperature sensing technologies that have implications in process monitoring and control in several industrial applications.

In a future work, we will consider an extensive study on the absolute area of the A-Scan to automate the measurement process of temperature measurement. Additionally, we will explore other liquids to further expand the scope of our methodology and consider an array of transducers for the acoustic tomographic reconstruction of temperature distribution.

## Figures and Tables

**Table 1:** Coefficients of polynomial equations describing speed of sound in relation to temperature, alongside standard deviations of experimental measurements.

Authors	$a_{0}$	$a_{1}$	$a_{2}$	$a_{3}$	$a_{4}$	$a_{5}$	Standard Deviation (mm/s)
Greenspan and Tschiegg [29]	$3.0449\times 10^{-9}$	$-1.45262\times 10^{-6}$	$3.31636\times 10^{-4}$	$-5.79506\times 10^{-2}$	5.03358	1402.736	26.3
Wilson [30]	0	$-8.248896\times 10^{-7}$	$2.8873\times 10^{-4}$	$-5.69215\times 10^{-2}$	5.02475	1403.013	160
Del Grosso and Mader [28]	$3.146\times 10^{-9}$	$-1.478\times 10^{-6}$	$3.342\times 10^{-4}$	$-5.80852\times 10^{-2}$	5.03711	1402.388	2.8

**Table 2:** Specifications of the H2KMPYA1000600 ultrasonic transducer from UNICTRON.

Specification	Value
Manufacturer	UNICTRON
Model	H2KMPYA1000600
Frequency	1.007 MHz *
Beam Angle	$5^{\circ }$
Applied Voltage	10 Vpp (Maximum 50 Vpp)
Minimal Sensitivity	−28 dB
Diameter	20.2 mm
Thickness	9.7 mm
Housing Material	Polyphenylene Sulfide
Sealing Material	Polyurethane
Attachment Method	Ethyl 2-cyanoacrylate Glue

* Frequency measured through experimental testing.

## Data Availability

Data are contained within the article.

## Abbreviations

The following abbreviations are used in this manuscript:

2D	Two-Dimensional
ADC	Analog-to-Digital Converter
AFE	Analog Front-End
BPF	Band-Pass Filter
FPAA	Field Programmable Analog Array
HPF	High-Pass Filter
MSPS	Mega Samples Per Second
MRI	Magnetic Resonance Imaging
PC	Personal Computer
PCB	Printed Circuit Board
PFT	Peak Flight Time
RTD	Resistance Temperature Detector
SNR	Signal-to-Noise Ratio
SPI	Serial Peripheral Interface
ToF	Time of Flight
UART	Universal Asynchronous Receiver-Transmitter
Vpp	Peak-to-Peak Voltage
Xducer	Transducer

## References

[1] Dutz F.J., Heinrich A., Bank R., Koch A.W., Roths J. (2019). Fiber-optic multipoint sensor system with low drift for the long-term monitoring of high-temperature distributions in chemical reactors. Sensors.

[2] Wang X., Zhang D., Wang X., Kong Z., Shao Y., Jin B. (2019). Thermal condition monitoring in a chemical looping combustion reactor for real-time operation diagnosis. Appl. Therm. Eng..

[3] García A., Toral V., Márquez Á., García A., Castillo E., Parrilla L., Morales D.P. (2018). Non-intrusive tank-filling sensor based on sound resonance. Electronics.

[4] Wahab Y.A., Rahim R.A., Rahiman M.H.F., Aw S.R., Yunus F.R.M., Goh C.L., Rahim H.A., Ling L.P. (2015). Non-invasive process tomography in chemical mixtures—A review. Sens. Actuators B Chem..

[5] Aloyan G., Kovalenko N., Grishchenko I., Konyashkin A., Ryabushkin O. (2022). Acoustic resonance spectroscopy of piezoelectric crystals under non-uniform heating. Acoust. Phys..

[6] Dukhin A.S., Goetz P.J. (2009). Bulk viscosity and compressibility measurement using acoustic spectroscopy. J. Chem. Phys..

[7] Holmes M., Parker N., Povey M. (2011). Temperature dependence of bulk viscosity in water using acoustic spectroscopy. Journal of Physics: Conference Series.

[8] Bagavathiappan S., Lahiri B.B., Saravanan T., Philip J., Jayakumar T. (2013). Infrared thermography for condition monitoring—A review. Infrared Phys. Technol..

[9] Liang H., Wang J., Zhang L., Liu J., Wang S. (2022). Review of optical fiber sensors for temperature, salinity, and pressure sensing and measurement in seawater. Sensors.

[10] Yapa S.D., D’Atri J.L., Schoech J.M., Elkins C.J., Eaton J.K. (2014). Comparison of magnetic resonance concentration measurements in water to temperature measurements in compressible air flows. Exp. Fluids.

[11] Pogány A., Wagner S., Werhahn O., Ebert V. (2015). Development and metrological characterization of a tunable diode laser absorption spectroscopy (TDLAS) spectrometer for simultaneous absolute measurement of carbon dioxide and water vapor. Appl. Spectrosc..

[12] Errigo M., Windows-Yule C., Materazzi M., Werner D., Lettieri P. (2023). Non-invasive and non-intrusive diagnostic techniques for gas-solid fluidized beds—A review. Powder Technol..

[13] Choe J.H., Lee K.S., Choy I., Cho W. (2018). Ultrasonic Distance Measurement Method by Using the Envelope Model of Received Signal Based on System Dynamic Model of Ultrasonic Transducers. J. Electr. Eng. Technol..

[14] Eckert S., Gerbeth G., Melnikov V. (2003). Velocity measurements at high temperatures by ultrasound Doppler velocimetry using an acoustic wave guide. Exp. Fluids.

[15] Sahoo A.K., Udgata S.K. (2019). A novel ANN-based adaptive ultrasonic measurement system for accurate water level monitoring. IEEE Trans. Instrum. Meas..

[16] Kokuryo D., Kumamoto E., Kuroda K. (2020). Recent technological advancements in thermometry. Adv. Drug Deliv. Rev..

[17] Chen Y., Qu M., Huang Y., Zheng Z., Cui P., Liu H., Xie J. (2023). Noninvasive measurement of temperature for simulated tissue based on piezoelectric micromachined ultrasonic transducers. J. Micromech. Microeng..

[18] Byra M., Klimonda Z., Kruglenko E., Gambin B. (2022). Unsupervised deep learning based approach to temperature monitoring in focused ultrasound treatment. Ultrasonics.

[19] Wang H., Zhou X., Yang Q., Chen J., Dong C., Zhao L. (2021). A reconstruction method of boiler furnace temperature distribution based on acoustic measurement. IEEE Trans. Instrum. Meas..

[20] Kong Q., Jiang G., Liu Y., Sun J. (2020). 3D high-quality temperature-field reconstruction method in furnace based on acoustic tomography. Appl. Therm. Eng..

[21] Zhong Q., Chen Y., Zhu B., Liao S., Shi K. (2022). A temperature field reconstruction method based on acoustic thermometry. Measurement.

[22] Liu Q., Zhou B., Zhang J., Cheng R., Dai M., Zhao X., Wang Y. (2023). A novel time-of-flight estimation method of acoustic signals for temperature and velocity measurement of gas medium. Exp. Therm. Fluid Sci..

[23] Schwarz M., Zagar B.G. (2022). Ultrasonic measurement and methods for reconstruction of temperature fields for the use in bioreactors. Tm-Tech. Mess..

[24] Lenner M., Kassubek F., Bernhard C., Yang L., Pape D. (2019). Single-Element Ultrasonic Transducer for Non-Invasive Measurements. IEEE Sens. J..

[25] Afaneh A., Alzebda S., Ivchenko V., Kalashnikov A. (2011). Ultrasonic measurements of temperature in aqueous solutions: Why and how. Phys. Res. Int..

[26] Zaz G., Calzavara Y., Le Clézio E., Despaux G. (2015). Adaptation of a high frequency ultrasonic transducer to the measurement of water temperature in a nuclear reactor. Phys. Procedia.

[27] Lubbers J., Graaff R. (1998). A simple and accurate formula for the sound velocity in water. Ultrasound Med. Biol..

[28] Del Grosso V., Mader C. (1972). Speed of sound in pure water. J. Acoust. Soc. Am..

[29] Greenspan M., Tschiegg C.E. (1959). Tables of the speed of sound in water. J. Acoust. Soc. Am..

[30] Wilson W.D. (1959). Speed of sound in distilled water as a function of temperature and pressure. J. Acoust. Soc. Am..

[31] Wong G.S., Zhu S.M. (1995). Speed of sound in seawater as a function of salinity, temperature, and pressure. J. Acoust. Soc. Am..

[32] Yu Y., Xiong Q., Ye Z.S., Liu X., Li Q., Wang K. (2022). A review on acoustic reconstruction of temperature profiles: From time measurement to reconstruction algorithm. IEEE Trans. Instrum. Meas..

